# The Natural Compound Homoharringtonine Presents Broad Antiviral Activity In Vitro and In Vivo

**DOI:** 10.3390/v10110601

**Published:** 2018-11-01

**Authors:** Hui-Jun Dong, Zhao-Hua Wang, Wen Meng, Cui-Cui Li, Yan-Xin Hu, Lei Zhou, Xiao-Jia Wang

**Affiliations:** Key Laboratory of Animal Epidemiology of the Ministry of Agriculture, College of Veterinary Medicine, China Agricultural University, Beijing 100193, China; donghuijun105@163.com (H.-J.D.); zhaohuaw26@163.com (Z.-H.W.); mengwen422fei@163.com (W.M.); piqiubang@163.com (C.-C.L.); huyx@cau.edu.cn (Y.-X.H.); leosj@cau.edu.cn (L.Z.)

**Keywords:** HHT, viral replication, RNA virus, DNA virus, eIF4E

## Abstract

To complement traditional antivirals, natural compounds that act via host targets and present high barriers to resistance are of increasing interest. In the work reported here, we detected that homoharringtonine (HHT) presents effective antiviral activity. HHT completely inhibited infections of vesicular stomatitis virus (VSV), Newcastle disease virus (NDV), and porcine epidemic diarrhea virus (PEDV) at concentrations of 50, 100, and 500 nM in cell cultures, respectively. Treatment with HHT at doses of 0.05 or 0.2 mg/kg significantly reduced viral load and relieved severe symptoms in PEDV- or NDV-infected animals. HHT treatment, however, moderately inhibited avian influenza virus (AIV) infection, suggesting its potent antiviral action is restricted to a number of classes of RNA viruses. In this study, we also observed that HHT actively inhibited herpes simplex virus type 1 (HSV-1) replication with a 50% inhibitory concentration (IC_50_) of 139 nM; the treatment with HHT at 1000 nM led to reductions of three orders of magnitude. Moreover, HHT antagonized the phosphorylation level of endogenous and exogenous eukaryotic initiation factor 4E (p-eIF4E), which might regulate the selective translation of specific messenger RNA (mRNA). HHT provides a starting point for further progress toward the clinical development of broad-spectrum antivirals.

## 1. Introduction

In recent decades, the understanding of the mechanism and regulation of translation made major progress. The translation process consists of three steps—initiation, elongation, and termination. The initiation phase can be carried out in two ways in eukaryotic cells—cap-dependent or cap-independent [1]. Some viruses accomplish translation initiation via cap-dependent mechanisms with varying utilization of host eukaryotic initiation factors (eIFs) [2]. In the cap-dependent mechanism, to translate a messenger RNA (mRNA) molecule, it is important that the mRNA be recruited in the eIF4F complex, which is composed of three proteins—the eIF4E protein, which binds to the cap, helicase eIF4A, and eIF4G [3,4]. The eIF4E protein can control eIF4F complex formation and interfere with cellular translation via phosphorylation on serine 209 [1,5]. The regulation of the elongation cycle is best studied in the eukaryotic system. All known viruses rely on cellular elongation factors for expression of the viral genome.

Viruses lack their own translational apparatus and are dependent entirely on the cellular translation machinery for their genome replication, new particle assembly, and release of new viral progeny. Especially for RNA viruses, translation of the viral genome is a critical control point in the replication cycle; several drugs were developed to take advantage of this vulnerability. As it is known, ribavirin of a guanosine analog inhibits replication of RNA and DNA viruses via distinctive mechanisms [6]. It represses the replication of vesicular stomatitis virus (VSV) via distinct mechanisms in different cell types, depending on intracellular ribavirin metabolism [7]. Among other mechanism of actions, ribavirin blocks eIF4E activity, and thus, reduces eIF4E-dependent survival signaling [8,9]. Silvestrol has multiple mechanisms of action, including an effect on certain microRNA, proteins important to protect the cell from damage, and proteins involved in the cell cycle [10]. It also acts as a specific inhibitor of eIF4A [11], which is a potent inhibitor of Ebola virus replication [12]. Viruses utilize host cell machinery of translation to facilitate their own replication. Inhibitors of translation elongation, an obligate step in the viral replication cycle, may also provide a general antiviral strategy. Properly used, cycloheximide (CHX) can block viral replication; unfortunately, its cytotoxic side effects and poor tolerability were prohibitive for its development as a drug [13,14]. Lactimidomycin (LTM) prevents the ribosome from leaving the start site and blocks the very first round of translation elongation. This recently established anticancer agent [15] potently inhibits infections of dengue virus and other RNA viruses in cell culture [16].

Homoharringtonine (HHT) is known to inhibit the first cycle of the elongation phase of eukaryotic translation [17,18]. HHT was used in China for the treatment of chronic myeloid leukemia (CML) and other types of tumor diseases for the past 30 years [19]. Omacetaxine, a semisynthetic form of HHT, was approved by the United States (US) Food and Drug Administration (FDA) for CML treatment in 2012. Pro-survival protein Mcl-1 and pro-oncoprotein c-Myc are critical regulators that promote the survival of leukemia cells; HHT could affect the survival of leukemia cells by allowing the rapid loss of short-lived Mcl-1 and c-Myc to facilitate proapoptotic triggering [19,20,21,22,23]. Leukemia cells express a high level of phosphorylated eIF4E; inhibiting phosphorylation of eIF4E represents a unique approach for the treatment of cancer development and progression [24,25]. It was recently reported that HHT targets the phosphorylated serine 209 residue of eIF4E, resulting in the degradation of phosphorylated eIF4E, thereby eradicating the growth of leukemia cells in vitro and in vivo [26,27].

In the present work, we observed that HHT represses the replication of viruses belonging to six families. HHT-treated embryos, chickens, pigs, and mice were found to be less susceptible to viral infections. Our preliminary results show that HHT antagonizes the level of phosphorylated eIF4E induced by viral infection. HHT offers the potential to be developed as a broad-spectrum antiviral.

## 2. Materials and Methods

### 2.1. Cells

Vero, HEK293T, and HeLa cells were cultured and maintained in Dulbecco’s modified Eagle’s medium (DMEM) supplemented with nonessential amino acids, 2 mM l-glutamine, sodium pyruvate, 5% or 10% heat-inactivated fetal bovine serum (FBS), 100 U/mL penicillin, and 100 mg/mL streptomycin (all reagents were purchased from Gibco Invitrogen, Carlsbad, CA, USA) in a humidified 37 °C, 5% CO_2_ incubator.

### 2.2. Viruses

Vesicular stomatitis virus (VSV) strain Indiana [28], porcine epidemic diarrhea virus (PEDV) strain CV777 [29], avian influenza virus (AIV) strain H9-WD [30], herpes simplex virus type 1 (HSV-1) strain F [31], and pseudorabies virus (PRV) strain Fa [32] were obtained according to the corresponding references indicated above. Newcastle disease virus (NDV) strain NA-1 was generated by inserting an additional transcription cassette coding for enhanced GFP between the P and M genes of the NDV genome (GFP-NDV) [33]. For in vitro antiviral assays, the cells were seeded at 2.5 × 10^6^ cells per well in standard six-well plates, after being infected with viruses for 1.5 h and resuspended in DMEM containing 2% FBS for indicated times.

### 2.3. Antibodies and Reagents

Mouse monoclonal anti-VSV-G (used at 1:1000) and HSV-ICP8 (1:1000) were obtained from Santa Cruz lnc. (Santa Cruz, CA, USA). The antibodies to actin (1:1000), goat anti-mouse secondary antibodies conjugated to horseradish peroxidase (HRP), and goat anti-rabbit secondary antibodies conjugated to HRP (1:10,000) were obtained from Beyotime Biotechnology (Haimen, China). The rabbit polyclonal antibody to phosphorylated (p)-eIF4E (Ser 209, 1:1000) was purchased from Cell Signaling Technology (Danvers, MA, USA). Rabbit polyclonal antibody to eIF4E (1:1000) was purchased from Proteintech (Wuhan, China). Mouse polyclonal antibody to PEDV-N (1:1000) was purchased from Alpha Diagnostic International (San Antonio, TX, USA). Rabbit polyclonal antibody to AIV-NP (1:2000) was purchased from Abcam (Cambridge, MA, USA). The antibodies to NDV-NP (1:2000) and PRV-gC (1:1000) were preserved in our laboratory. Antibodies were diluted in phosphate-buffered saline (PBS) containing 5% skim milk (pH 7.3). Ribavirin, acyclovir, quercetin, and the mitogen-activated protein kinase (MAPK)-interacting kinase 1 and 2 (MNK1/2) kinase inhibitors CGP57380 were obtained from MedChemExpress (Monmouth Junction, NJ, USA).

### 2.4. Plaque Formation Assay

Vero cells seeded at 2.5 × 10^5^ cells per well in 12-well plates were infected with ten-fold serially diluted HSV-1 for 1.5 h. Then, infected cells were incubated at 37 °C in DMEM containing 1% FBS and 0.5% low-melt point agarose for 72 h. The agarose was removed by suction and stained with 0.5% crystal violet in ethanol. Plaque numbers were counted. Data represent the mean of three independent experiments.

### 2.5. Determination of Half Maximal Tissue Culture Infective Dose (TCID_50_)

Cells were mock infected or exposed to virus in a mixture supplemented with DMEM. After 1.5 h, the inoculum was replaced with DMEM containing 2% FBS and incubated for the indicated times. Virus titration was performed in cells seeded on 96-well plates at 10^4^ cells/well. Ten-fold serial dilutions were prepared for each sample and 100 µL/well of each dilution was added to the cells in quadruplicates. When the cytopathic effect stabilized, cells were analyzed using light microscopy and the log_10_ TCID_50_/mL was determined using the Reed–Muench method.

### 2.6. Time-of-Addition Study

For the pre-treatment assay, cell monolayers were treated with HHT at a concentration of 50 nM (for VSV) or 400 nM (for PEDV) for 2 h at 37 °C, prior to being washed twice with PBS and infected with virus at a multiplicity of infection (MOI) of 0.5 (for VSV) or 0.1 (for PEDV). After the 1.5-h virus adsorption period, infected cells were incubated at 37 °C in DMEM supplemented with 2% FBS for 48 h before viral yields in the medium were determined by TCID_50_. For the co-treatment and post-treatment assays, cells were infected with virus for 1.5 h (adsorption), and HHT was added at different time points. Infected cells were harvested at 48 h post infection for quantification of viral yield.

### 2.7. Preparation of Cell Lysates and Western Blotting

The cells were collected by centrifugation and dissolved in 200 µL of lysis buffer in the presence of the protease inhibitor cocktail and finally disrupted by sonication. The cell suspension was then fractionated by centrifugation at 6000× *g* for 20 min at 4 °C. Solubilized proteins were harvested, electrophoresed in denaturing polyacrylamide gels, electroblotted onto a polyvinylidene fluoride (PVDF) membrane, and reacted with the antibodies indicated. Protein bands were detected with secondary antibody conjugated to horseradish peroxidase (HRP) for 45 min at room temperature, and actin was used as a loading control.

### 2.8. Quantitative Real-Time PCR (qRT-PCR)

Replicated cultures were harvested and total RNA was extracted using Trizol reagent (Invitrogen) according to the manufacturer’s protocol. A two-step RT-PCR (SYBR Green I technology, Applied Roche Diagnostics, Mannheim, Germany) was performed using SYBR green supermix (Toyobo, Osaka, Japan) according to the manufacturer’s protocol to measure transcription levels for several genes of interest. The primers used were as follows: NDV-NP, 5′–TTT TGC TAA CAG TGT GCC CC–3′ (forward), 5′–ATC TTC AAC CCC AGC TGT GA–3′ (reverse); PEDV-N, 5′–CTG GGT TGC TAA AGA AGG CG–3′ (forward), 5′–CTG GGG AGC TGT TGA GAG AA–3′ (reverse); actin, 5′–CGT TGA CAT CCG TAA AGA CC–3′ (forward), 5′–CTA GGA GCC AGA GCA GTA ATC–3′ (reverse); glyceraldehyde 3-phosphate dehydrogenase (GAPDH): 5′–GAT CAT CAG CAA TGC CTC CT–3′ (forward), 5′–TGA GTC CTT CCA CGA TAC CA–3′ (reverse). Relative fold changes were automatically calculated by the Step One Plus real-time PCR system software (Applied Biosystems, Foster City, CA, USA), following the ∆∆C_T_ method. Actin was also determined and used as internal control.

### 2.9. Specific Pathogen-Free (SPF) Chicken Embryo Assay

For each inoculation, a mixture of HHT (Aladdin, 98% purity) and NDV (50 plaque-forming units (PFU)) or AIV (500 PFU) was injected into the allantoic cavity of specific pathogen-free (SPF) chicken eggs. Eggs were incubated for different times, and allantoic fluid was harvested to measure viral yields, as described in our previous report [34].

### 2.10. Hemagglutination (HA) Assay

Each chicken embryo allantoic fluid was harvested and two-fold serially diluted in sterile saline; each dilution of 25 μL was mixed with an equal volume of 1% washed red blood cells (RBC) of chicken. The maximum dilution of allantoic fluid that still resulted in complete agglutination of RBC suspension was recorded as HA unit (HAU) of virus titer.

### 2.11. In Vivo Antiviral Assays

SPF chickens were challenged by intramuscular injection with 10^4^ PFU of GFP-NDV, and were treated with 0.2 mg/kg HHT or PBS for three days. NDV-NP mRNA in the liver and lung was quantified by qRT-PCR at seven days post infection. SPF piglets were injected intramuscularly with 2 × 10^3^ PFU of PEDV and 0.05 mg/kg HHT for three sequential days. PEDV-N mRNA in intestine was quantified by qRT-PCR at five days post infection. Total RNA was prepared from 10 mg of tissue homogenized in Trizol according to the manufacturer’s instructions. The DNaseI-treated RNA (0.2 μg) was reverse-transcribed into complementary DNA (cDNA). A two-step RT-PCR (SYBR Green I technology, Applied Roche) was performed using SYBR green supermix (Toyobo) according to the manufacturer’s protocol. Mice were intranasally injected with 10^6^ PFU of AIV, and were intraperitoneally injected with 0.8 mg/kg HHT for two sequential days. Representative lung sections from each group were subjected to immunohistochemical analysis and hematoxylin and eosin (H&E) staining at two or four days post infection. Animals were observed daily for clinical signs. The animals were euthanized by injecting pentobarbital sodium intravenously. To reduce the stress to other animals, euthanasia was carried out in a soundproof room to avoid panic of the living animals. Animal protocols approved by the Animal Welfare Committee of China Agricultural University were followed and the animals were housed with pathogen-free food and water under 12-h light-cycle conditions.

### 2.12. Immunohistochemical Analysis and H&E Staining

The AIV-infected mice indicated above were sacrificed at the indicated days post infection, and their lungs were then harvested and fixed in 10% neutral buffered formalin. Organs were then paraffin-embedded, sectioned, and stained with hematoxylin and eosin and subjected to immunohistochemical analysis using antibodies to AIV-NP. The expression of nucleoprotein (NP) was semi-quantitatively analyzed under a light microscope (magnification 40×). The histopathology of the images was observed under a light microscope (magnification 20×). Pathological changes were observed under an Olympus microscope (BX41; Olympus, Tokyo, Japan).

### 2.13. Plasmids

The vector pcDNA3.1 was purchased from Clontech (Mountain View, CA, USA). FLAG-tagged eIF4E and FLAG-tagged eIF4E-S209A mutant were cloned in a vector using the design primers. Eukaryotic expression plasmids FLAG-tagged eIF4E and FLAG-tagged eIF4E-S209A mutant were transfected into cells with the aid of Lipofectamine LTX (Invitrogen) according to the manufacturer’s instructions.

### 2.14. 3-(4,5-Dimethylthiazol-2-yl)-2,5-diphenyltetrazolium Bromide (MTT) Assay

The MTT assay was conducted according to the manufacturer’s (Beyotime Biotechnology) protocol. Briefly, cells in 96-well plates were treated with increasing doses of HHT at 37 °C and 5% CO_2_ for 24 h. Then, 10 µL of MTT solution was added to each well and the samples were incubated for 4 h. The medium was then removed and 100 µL of Formazan solution was added to each well. Optical density (OD) values were measured at a test wave length of 570 nm using a Multiskan Ascent Microplate Photometer (Thermo Fisher Scientific, Waltham, MA, USA).

### 2.15. Statistics

All results were expressed as means and standard deviations (SD). Statistical analyses were performed using Prism 5.01 (GraphPad Software, La Jolla, CA, USA). Significance was determined by one-way analysis of variance (ANOVA) and two-way ANOVA with Dunnett’s multiple-comparison test. Partial correlation analyses were evaluated using an unpaired Student’s *t*-test.

### 2.16. Ethics Statement

All animal research projects were sanctioned by the Beijing Laboratory Animal Welfare and Ethics Committee and were approved by the Animal Ethics Committee of China Agricultural University (approval number 201206078) and were performed in accordance with Regulations of Experimental Animals of Beijing Authority.

## 3. Results

### 3.1. HHT Inhibits the Late Stage of VSV Replication

**Figure 1:**
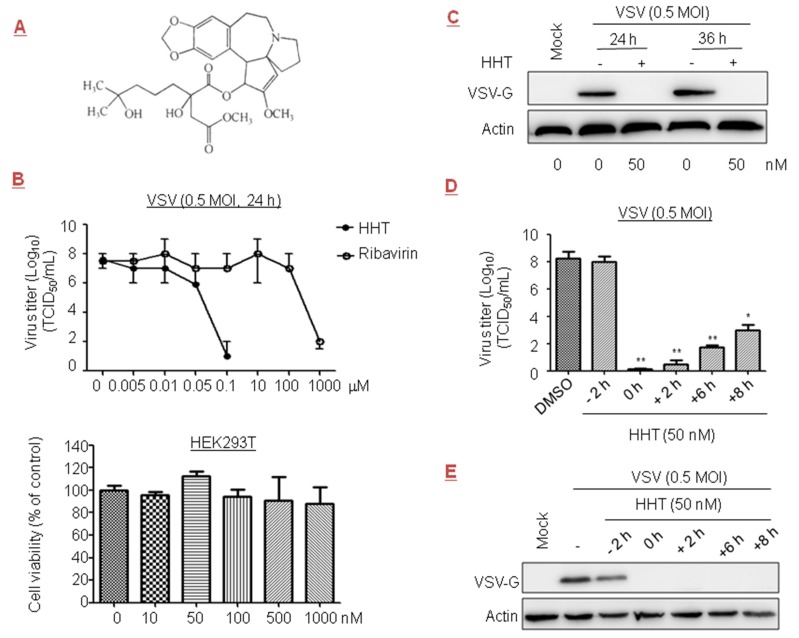
Inhibitory effect of homoharringtonine (HHT) on vesicular stomatitis virus (VSV) infection. (**A**) Chemical structure of HHT. (**B**) Dose-dependent studies. HEK293T cells seeded at 2.5 × 10^6^ cells per well in standard six-well plates were infected with VSV at 0.5 multiplicity of infection (MOI) in the presence of HHT or ribavirin at different concentrations for 24 h. HHT and ribavirin were dissolved in phosphate-buffered saline (PBS) containing 1% dimethyl sulfoxide (DMSO). Viral yields in the medium were determined by half maximal tissue culture infective dose (TCID_50_). These experiments were performed two times with three replicates in each experiment. Values represent means and standard deviation (SD). Bottom, effects of HHT on cell viability. HEK293T cells were treated with various concentrations of HHT for 24 h, and cell viability was determined by 3-(4,5-dimethylthiazol-2-yl)-2,5-diphenyltetrazolium bromide (MTT) assay. These experiments were performed two times with three replicates in each experiment. Values represent means and SD. (**C**) HEK293T cells were mock infected or infected with VSV at 0.5 MOI in the presence of PBS or HHT of 50 nM. At 24 and 36 h post infection (h.p.i.), cell lysates were harvested and electrophoretically separated proteins were analyzed by immunoblotting with antibody to viral protein VSV-G, with actin as a control. (**D**,**E**) Time-of-addition study. HEK293T cells were exposed to VSV at 0.5 MOI, and treated with HHT at 50 nM at different time points before, simultaneously with, or after infection. At 48 h.p.i., viral yields in the medium were determined by TCID_50_. These experiments were performed two times with three replicates in each experiment. Values represent means and SD. Statistical analysis was evaluated by one-way analysis of variance with Dunnett’s multiple-comparison test. * *p* < 0.05; ** *p* < 0.01 compared to PBS-treated group (**D**). Under the same experimental conditions, electrophoretically separated proteins were analyzed by immunoblotting with antibodies to VSV-G and actin (**E**).

The prototype rhabdovirus VSV is widely studied as a model of viral protein translational control [35]. Early reports showed that ribavirin at 800 µM inhibited VSV titer in cells by 2.5 orders of magnitude [36]. In this paper, we evaluated the effect of a plant alkaloid HHT (Figure 1A), a natural product first discovered in *Cephalotaxus harringtonii*, on VSV replication. No measurable decrease in cell viability was detected at a concentration of 1 µM [37,38]. Therefore, HHT at concentrations below 1 µM was administered in the experiments described here. For testing potential dose-dependent antiviral activity, HEK293T cells were infected with VSV in the presence of HHT or ribavirin at increasing concentrations. We observed that viral yields were reduced by 1.5 orders of magnitude when treated with HHT at 50 nM, and viral yields were dramatically reduced in cells treated with HHT at 100 nM; no measurable decrease in cell viability was detected. Ribavirin displayed antiviral activity at concentrations over 100 µM (Figure 1B).

To analyze the specificity of HHT for viral replication, we investigated its impact on the level of viral protein by Western blotting. We observed a strong reduction in viral protein G levels of VSV (VSV-G) at 24 and 36 h post infection (h.p.i.) under treatment with HHT at 50 nM (Figure 1C). Time-of-addition studies were conducted to identify the window in the VSV replication cycle when HHT exerted its antiviral effect. The results showed that pre-treatment of cells with HHT for 2 h prior to VSV infection produced a minimal inhibitory effect against viral infection. This suggests that HHT does not inhibit the VSV entry process (Figure 1D). A complete reduction of the viral yields was observed when HHT was added at the time of infection and from 2 h.p.i. onward. Addition of HHT at 6 and 8 h.p.i. led to strong viral reductions of 6.5 and 5 orders of magnitude. Under the same experimental conditions, strong reductions in VSV-G levels were observed with co-treatment and post-treatment for up to 8 h for VSV infection (Figure 1E). These results indicate that HHT acts in the viral replication cycle after viral entry.

### 3.2. HHT Reduces Viral Load in NDV-Infected Cells, Embryos, and Chickens

**Figure 2:**
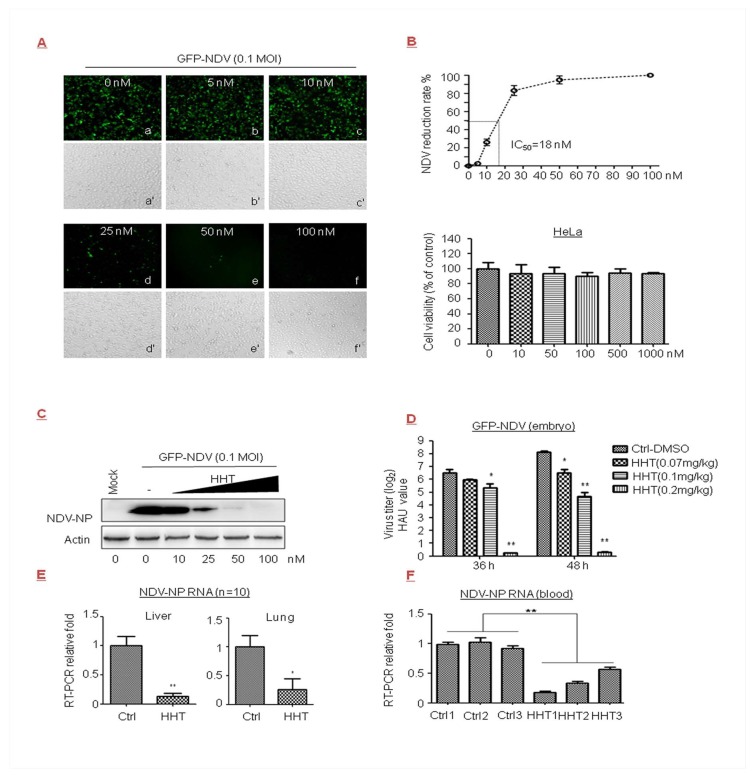
Inhibitory effect of HHT on Newcastle disease virus (NDV) infection in cells, embryos, and chickens. (**A**–**C**) Dose-dependent studies. HeLa cells seeded at 2.5 × 10^5^ cells per well in 24-well plates were exposed to GFP-NDV at 0.1 MOI in the presence of HHT at increasing concentrations for 24 h. Expression of GFP was directly observed using a DP73 camera by fluorescence microscopy on an Olympus IX73 microscope 25 (magnification 10×) (**a**–**f**), and were assessed under light microscopy (**a′**–**f′**). One image is representative of three (**A**). Under the same experimental conditions, the infection rate of HHT on GFP-NDV infection was expressed as percent reduction in fluorescence activity of the infected cells without HHT treatment, which was set at 0%. Half maximal inhibitory concentration (IC_50_) was calculated as indicated by the fine dotted lines. These experiments were performed two times with three replicates in each experiment. Values represent means and SD. Bottom, effects of HHT on HeLa cell viability were determined, and assessed as indicated in Figure 1B. Statistical analysis was evaluated by one-way analysis of variance with Dunnett’s multiple-comparison test. * *p* < 0.05 compared to control group (**B**). HeLa cells were seeded at 2.5 × 10^6^ cells per well in six-well plates. Under the same experimental conditions, cell lysates were harvested and electrophoretically separated proteins were analyzed by immunoblotting with antibody to viral protein NDV-NP, with actin as a control (**C**). (**D**) Inhibitory effect of HHT on NDV infection in chicken embryos. HHT at different concentrations or PBS in 100 µL volume was injected into the allantoic cavity of 11-day specific pathogen-free (SPF) chicken eggs simultaneously with GFP-NDV inoculation of 50 plaque-forming units (PFU). The eggs were incubated for 36 and 48 h and analyzed at the same time. Viral yields in allantoic fluids were determined as log_2_ hemagglutination units (HAU)/50 µL. Graphs represent the average from three embryos per experimental group, and error bars denote standard errors of the means. Each experiment was performed in triplicate. Statistical analysis was evaluated by two-way analysis of variance with Dunnett’s multiple-comparison test. * *p* < 0.05; ** *p* < 0.01 compared to PBS-treated eggs. (**E**,**F**) In vivo antiviral efficacy. Four- to five-week-old SPF chickens were challenged via intramuscular (i.m.) routes with 10^4^ PFU of GFP-NDV, and simultaneously with 0.2 mg/kg/day HHT or PBS containing 1% DMSO in 100 µL volume for three days, at one-day intervals. NDV-NP messenger RNA (mRNA) in the liver and lung was quantified by qRT-PCR at seven days post infection. Graphs represent the average from 10 animals per experimental group, and values represent means and SD. These experiments were performed two times with three replicates in each experiment. Values represent means and SD. Statistical analysis was evaluated by one-way analysis of variance with Dunnett’s multiple-comparison test. * *p* < 0.05; ** *p* < 0.01 compared to PBS-treated eggs (**E**). Under the same experimental conditions, NDV-NP mRNA in three randomly drawn blood samples were analyzed by qRT-PCR. Each group represents independent experiments. Each experiment was performed in triplicate. Statistical analysis was evaluated using an unpaired Student’s *t*-test; ** *p* < 0.01 compared to PBS-treated chickens (**F**).

Newcastle disease of the Paramyxoviridae family is one of the most important avian diseases in poultry [39]. In the present research effort, cells were infected with GFP-NDV in the presence of increasing doses of HHT. We observed that HHT at 50 nM greatly reduced the production of a recombinant NDV expressing green fluorescent protein (GFP), as determined by light microscopy (Figure 2A). HeLa cells displayed no difference in morphology and number compared to the mock infected sample when treated with HHT at 100 nM. Correspondingly, HHT treatment showed a dose-dependent reduction in infection rate. As shown in Figure 2B, we estimated a 50% inhibitory concentration (IC_50_) value of 18 nM, and an IC_90_ of 40 nM was observed. No measurable decrease in cell viability was detected at concentrations below 1 µM (Figure 2B). We also observed a strong reduction in NDV-NP level in cells treated with HHT at 50 nM (Figure 2C).

We further examined the ability of HHT to reduce GFP-NDV replication in chicken embryos. This is a widely used in vivo model of viral infection [34,40]. GFP-NDV and different doses of HHT were injected into the allantoic cavity of SPF chicken embryos. Viral yields from allantoic fluids of embryos were measured by HA assay. As shown as in Figure 2D, HHT at a dose of 0.07 mg/kg had a slight effect on viral infection; 0.1 mg/kg HHT reduced HAU values by one and three orders of magnitude at 36 and 48 h.p.i., respectively. Remarkably, 0.2 mg/kg HHT provided the optimal inhibitory activity, and completely inhibited viral infection. Finally, we studied whether HHT displays potent antiviral activity against NDV in chickens. As shown in Figure 2E,F, 0.2 mg/kg HHT significantly decreased the mRNA level of NDV-NP in the liver and lung (Figure 2E), and blood (Figure 2F). HHT-treated animals did not exhibit a pathological change in tissues or symptoms such as diarrhea and drooping.

Our results indicated that HHT effectively reduces viral load in NDV-infected cells, embryos, and chickens.

In this study, we also observed that ribavirin inhibited GFP-NDV replication with an IC_50_ value of 44.241 µM (Table 1). IC_50_ and half maximal cytotoxic concentration (CC_50_), as well as the selectivity index value, showed that the HHT treatment at the indicated dosages was more effective than ribavirin, without affecting cell viability.

### 3.3. HHT Reduces Viral Load in PEDV-Infected Cells and Piglets

**Figure 3:**
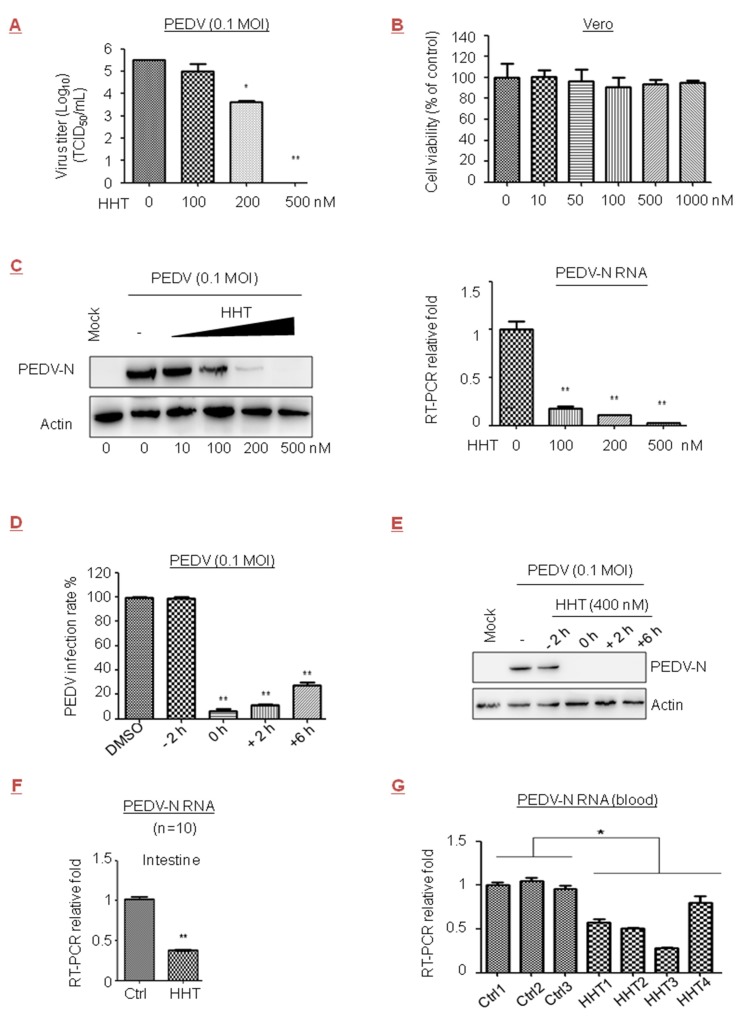
Inhibitory effect of HHT on porcine epidemic diarrhea virus (PEDV) infection in cells and piglets. (**A**–**C**) Dose-dependent studies. Vero cells seeded at 2.5 × 10^6^ cells per well in six-well plates were infected with PEDV at 0.1 MOI in the presence of the HHT at different concentrations for 48 h. Viral yields in the medium were determined by TCID_50_. These experiments were performed two times with three replicates in each experiment. Values represent means and SD. Statistical analysis was evaluated by one-way analysis of variance with Dunnett’s multiple-comparison test. * *p* < 0.05; ** *p* < 0.01 compared to PBS-treated group (**A**). Effects of HHT on Vero cell viability were determined, and assessed as indicated in Figure 1B (**B**). Under the same experimental conditions, electrophoretically separated proteins were analyzed by immunoblotting with antibodies to viral protein PEDV-N and actin. Bottom, total RNA was isolated, and PEDV-N mRNA was quantified by qRT-PCR normalized against actin. Statistical analysis was evaluated by one-way analysis of variance with Dunnett’s multiple-comparison test. ** *p* < 0.01 compared to PBS-treated group (**C**). (**D**,**E**) Time-of-addition study. Vero cells were treated with HHT at 400 nM at different time points before and after infection. At 48 h.p.i., the infection rate of HHT on PEDV infection was expressed as a percentage of the infected cells without HHT treatment, which was set at 100%. These experiments were performed two times with three replicates in each experiment. Values represent means and SD. Statistical analysis was evaluated by one-way analysis of variance with Dunnett’s multiple-comparison test. ** *p* < 0.01 compared to PBS-treated group (**D**). Under the same experimental conditions, cells were harvested and electrophoretically separated proteins were analyzed by immunoblotting with antibodies to PEDV-N and actin (**E**). (**F**,**G**) In vivo antiviral efficacy. Three- to five-day-old SPF piglets were challenged via intramuscular (i.m.) injection with 2 × 10^3^ PFU of PEDV, and at the same time were treated with 0.05 mg/kg/day HHT or PBS containing 1% DMSO in 100 µL volume for three sequential days. PEDV-N mRNA in intestine was quantified by qRT-PCR at five days post infection. Graphs represent average from 10 animals per experimental group. Values represent means and SD. These experiments were performed two times with three replicates in each experiment. Statistical analysis was evaluated by one-way analysis of variance with Dunnett’s multiple-comparison test. ** *p* < 0.01 compared to PBS-treated piglets (**F**). Under the same experimental conditions, PEDV-N mRNA in three to four randomly drawn blood samples were also analyzed by qRT-PCR, and each group represents independent experiments. Each experiment was performed in triplicate. Statistical analysis was evaluated using an unpaired Student’s *t*-test; * *p* < 0.05 compared to PBS-treated piglets (**G**).

PEDV of the Coronaviridae family causes viremia and high mortality rates in newborn piglets. The efficacy of the available commercial vaccines is limited or the protective immunity is insufficient [41,42]. We observed that viral yields were reduced by 1.9 orders of magnitude under treatment with HHT at 200 nM in Vero cells; they were completely suppressed after treatment with HHT at 500 nM (Figure 3A). No measurable decrease in cell viability was detected at a concentration of 1 µM (Figure 3B). Quercetin, a natural compound, affects the initial stage of PEDV infection by interfering with viral replication [43]. The IC_50_ values of HHT and quercetin in PEDV were 0.112 µM and 6.897 µM, respectively (Table 1). A strong reduction in PEDV-N protein and mRNA levels under treatment of cells with HHT was also observed (Figure 3C). HHT at 400 nM was added at different times before and after infection of cells with PEDV. Similar to the result shown in Figure 1D, a less than 30% infection rate was found when HHT was added at 0, 2, and 6 h.p.i. (Figure 3D), and strong reductions in PEDV-N levels were also observed (Figure 3E).

We further examined the ability of HHT to reduce PEDV infection in piglets, and found that treatment with 0.2 mg/kg of HHT led to severe side effects, fatal to about 50% of the animals. HHT at a dose of 0.05 mg/kg provided optimal inhibitory activity without death and was administered in subsequent experiments. We detected that HHT treatment greatly decreaseed mRNA levels of PEDV-N in the intestine (Figure 3F) and blood (Figure 3G). HHT-treated animals did not exhibit pathological change in tissues or symptoms of diarrhea and cachexia.

Our results indicated that HHT effectively reduces viral load in PEDV infected cells and animals.

### 3.4. HHT Exhibits Antiviral Activity against AIV

**Figure 4:**
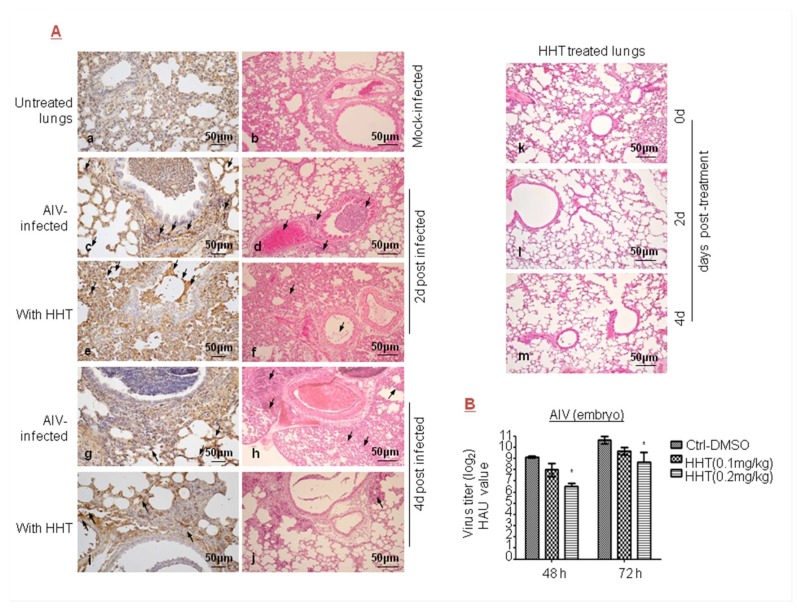
Inhibitory effect of HHT on avian influenza virus (AIV) infections. (**A**) Antiviral activity of HHT against AIV. Six- to eight-week-old BALB/c mice were mock infected (**a**,**b**) or intranasally (i.n.) injected with 10^6^ PFU of AIV, while being intraperitoneally (i.p.) injected with 0.8 mg/kg/day HHT (**e**,**f**,**i**,**j**) or PBS containing 1% DMSO (**c**,**d**,**g**,**h**) in 100 µL volume for two sequential days. Representative lung sections from each group were subjected to immunohistochemical analysis with antibody against AIV-NP (left) and hematoxylin and eosin (H&E) staining (right) at two or four days post infection. HHT at days 0, 2, and 4 after treatment (**k**,**l**,**m**). Arrows in right panel indicate inflammatory cell infiltration, erythrocyte infiltration, and drop in mucous epithelium in bronchia (**A**). SPF chicken egg was challenged with AIV inoculation of 500 PFU, and simultaneously treated with 0.1 or 0.2 mg/kg HHT or PBS containing 1% DMSO. The eggs were incubated for 48 and 72 h and analyzed at the same time, and assessed as described in Figure 2D. * *p* < 0.05, compared to DMSO-treated eggs (**B**).

To analyze the antiviral activity of HHT on AIV of the Orthomyxoviridae family, we examined the ability of HHT to reduce AIV replication in mice. Using immunohistochemistry analysis and H&E staining, we detected the distribution of AIV (Figure 4Ac,g) and an inflammation and erythrocyte infiltration (d,h) in the lungs of infected mice. No obvious reduction in the number of AIV positive cells was observed in mice treated with HHT at a dose of 0.8 mg/kg (e,i). Moreover, HHT treatment for two days decreased local inflammatory reaction but aggravated overall inflammation (f), while HHT treatment for four days resulted in moderately attenuated inflammation (j). No obvious inflammatory reaction was seen in mice treated with HHT at days 0, 2, and 4 after administration (k,l,m). These results indicated that HHT did not efficiently affect AIV-induced inflammation and damage. We also studied the effect of HHT on AIV in embryos. As shown in Figure 4B, 0.1 mg/kg HHT reduced the HAU value by 1.1 and 1.3 orders of magnitude at 48 and 72 h.p.i.; and 0.2 mg/kg HHT reduced the HAU value by 2.6 and 2.0 orders of magnitude at 48 and 72 h.p.i., respectively. Taken together, we conclude that, for a given dose, HHT did not produce inhibitory effects on AIV, as compared to its action on other RNA viruses considered in this paper.

### 3.5. HHT Presents Dose-Dependent Inhibition of Herpes Virus Infections

**Figure 5:**
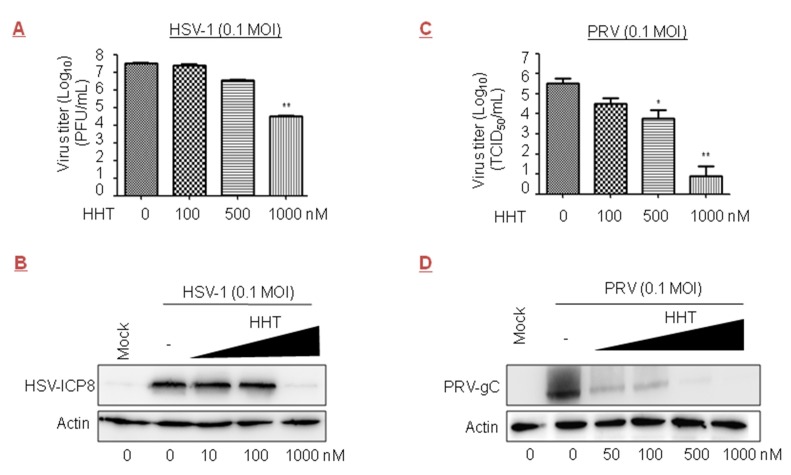
Inhibitory effect of HHT on herpes simplex virus type 1 (HSV-1) and pseudorabies virus (PRV) infections. (**A**,**B**) Antiviral activity of HHT against HSV-1. Vero cells seeded at 2.5 × 10^6^ cells per well in six-well plates were infected with HSV-1 at 0.1 MOI in the presence of HHT at increasing concentrations. At 72 h.p.i., viral yields were determined by plaque assay and are presented as log_10_ PFU/mL. These experiments were performed two times with three replicates in each experiment. Values represent means and SD. Statistical analysis was evaluated by one-way analysis of variance with Dunnett’s multiple-comparison test. ** *p* < 0.01 compared to PBS-treated group (**A**). At 48 h.p.i., cell lysates were harvested and electrophoretically separated proteins were analyzed by immunoblotting with antibodies to viral protein HSV-ICP8 and actin (**B**). (**C**,**D**) Antiviral activity of HHT against PRV. Vero cells were infected with PRV at 0.1 MOI in the presence of HHT at increasing concentrations as indicated. At 48 h.p.i., viral yields in the medium were determined by TCID_50_, and assessed as indicated in Figure 3A. * *p* < 0.05; ** *p* < 0.01 compared to PBS-treated group (**C**). At 36 h.p.i., cell lysates were harvested and electrophoretically separated proteins were analyzed by immunoblotting with antibodies to viral protein PRV-gC and actin (**D**).

We tested the inhibitory effect of HHT on the DNA viruses HSV-1 and PRV, both members of the Herpesviridae family. We observed that treatment with HHT at 500 and 1000 nM led to reductions of one and three orders of magnitude in Vero cells, respectively (Figure 5A); a strong reduction in HSV-ICP8 level was also observed (Figure 5B). Acyclovir, an inhibitor of viral DNA replication, is the only approved medicine for HSV-1 infection therapies [44,45]. The IC_50_ values of HHT and acyclovir in HSV-1 were 139 nM and 789 nM, respectively (Table 1). We also found that HHT at 100, 500, and 1000 nM produced inhibitions of viral yield of 1.0, 1.8, and 4.6 orders of magnitude in Vero cells, respectively, for PRV (Figure 5C). HHT treatment at 500 nM led to a strong reduction in PRV-gC level (Figure 5D). There were obvious differences in the ability of HHT to reduce the viral replication of HSV-1 and PRV.

### 3.6. HHT Reduces the Level of Phosphorylated eIF4E

**Figure 6:**
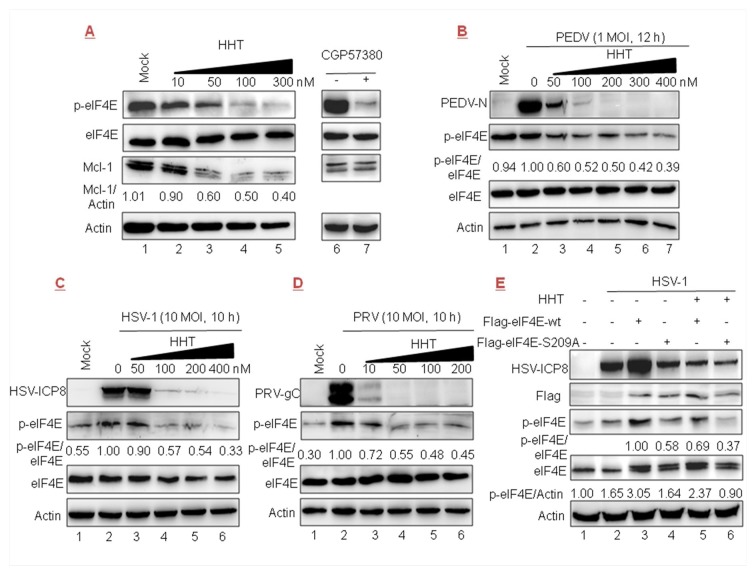
HHT regulates the phosphorylation levels of eukaryotic initiation factor 4E (eIF4E). (**A**) Vero cells seeded at 2.5 × 10^6^ cells per well in six-well plates were treated with increasing doses of HHT for 24 h. Electrophoretically separated proteins were analyzed by Western blotting using antibodies directed against phosphorylated eIF4E (p-eIF4E), eIF4E, and Mcl-1, with actin as a control. (**B**–**D**) Vero cells were mock infected (lane 1) or infected with PEDV (panel **B**), HSV-1 (panel **C**), or PRV (panel **D**) for 12, 10, or 10 h (lanes 2–6) in the presence of HHT at different concentrations. Electrophoretically separated proteins were analyzed by Western blotting using antibodies directed against p-eIF4E, eIF4E, PEDV-N, HSV-ICP8, PRV-gC, and actin. Densitometry analyses were quantified using the ImageJ software. (**E**) HeLa cells in a T-25 flask were transfected with FLAG-tagged eIF4E (lanes 3,5) or eIF4E-S209A plasmid (lanes 4,6) (5 μg each), in the absence (lanes 1–4) or presence (lanes 5,6) of HHT at 100 nM, then mock infected (lane 1) or infected with HSV-1/F at 10 MOI for 10 h (lanes 2–6). Cell lysates were then analyzed by Western blotting with the indicated antibodies. One of two independent experiments is shown.

It was reported that HHT reduces the phosphorylation level of eIF4E (p-eIF4E), as well as the level of Mcl-1 [22,26]. In the present work we detected that the accumulation of Mcl-1 was gradually reduced in cells treated with increasing doses of HHT (Figure 6A, lanes 3–5). We also found that p-eIF4E levels were gradually reduced (lanes 3–5), while cellular total eIF4E level was unaffected. The positive control was treated with CGP57380 (inhibitor of MNK1/2 kinase, the eIF4E upstream kinase) [46], which can prevent the phosphorylation of eIF4E (at Ser 209) without an effect on total eIF4E protein level (lane 7).

We further explored whether the inhibitory effect of HHT on viral infections is related to the phosphorylation of eIF4E. The results showed that the level of endogenous p-eIF4E was increased upon PEDV, HSV-1, and PRV (Figure 6B–D, lanes 1–2) infection. Furthermore, high levels of p-eIF4E, in parallel with the level of viral protein, were gradually reduced under treatment with increasing doses of HHT (lanes 3–7). These results demonstrate that the antiviral effect of HHT might be associated with its function as a p-eIF4E inhibitor. To further clarify whether HHT regulating p-eIF4E level is associated with viral replication, two constructs encoding the wild-type FLAG-tagged eIF4E and the phosphorylation-incompetent variant eIF4E-S209A were transiently transfected, followed by HSV-1 infection. As shown in Figure 6E, the ectopic expression of wild-type eIF4E (lane 3), but not eIF4E-S209A (lane 4), greatly improved the level of p-eIF4E, and also upregulated the expression of viral ICP8 (lane 3). Furthermore, we detected that HHT treatment decreased the accumulation of p-eIF4E induced by the ectopic expression of wild-type eIF4E, coincident with decreased viral propagation (lane 5). Taken together, the results shown in Figure 6B–E indicate that HHT attenuates the phosphorylation level of endogenous and exogenous eIF4E to counteract HSV-1 replication.

## 4. Discussion

The high virulence of viruses and the absence of effective therapies pose an ongoing threat to public health. Due to the intrinsically high mutation rate of RNA viruses, resistance to antiviral drugs that act against viral targets can occur rapidly [47]. Focus on antivirals that target host factors is, thus, expected to be an advantageous strategy, because escape mutations are rarer [48,49]. Cellular translation is necessary for viral replication, and inhibition of protein production may impair or delay the proliferation of viral pathogens; however, the inhibition of host factors is often a problematic issue in drug development, due to pleiotropic unwanted side effects [50,51].

HHT at a high dose (1.0 mg/kg) was found in previous work to completely eradicate acute myeloid leukemia in vivo in mice, and all the mice obtained long-term disease-free survival [26]. In fact, data from patients without viral infections suggest that HHT is generally safe [18]. The in vivo effects and cytotoxicity of HHT in piglets, chicken embryos, and chickens was not previously reported. In our study, HHT at doses of 0.05 mg/kg for piglets and 0.2 mg/kg for chickens effectively repressed viral production. All the treated animals survived without pathogenic changes in tissues and symptoms. In addition, HHT displayed a 50% toxicity dose when piglets and chickens were treated with 0.2 and 0.4 mg/kg, respectively. HHT provides a promising starting point for advancing further toward the clinical development of antivirals.

The antiviral efficacy of HHT on hepatitis B virus (HBV) DNA levels and bovine viral diarrhea virus (BVDV) infection were previously described, with HHT at concentrations below 2 µM having no protective effects [52]. HHT at 1 µM produced an inhibition of viral yield of 2–3 orders of magnitude for chikungunya virus [38]. Treatment with HHT at 125 nM was recently shown to produce high-magnitude inhibition for mouse hepatitis coronavirus (MHV) in vitro by inhibiting viral translation [53]. In the current study, we detected that HHT presents broad antiviral activity in host cells. HHT completely inhibited the infection of RNA viruses VSV, NDV, and PEDV at 50, 100, and 500 nM in cell cultures, respectively; a time-of-addition study revealed that HHT remains potent even when administrated 8 h post infection. By comparison, HHT treatment only moderately inhibited the infection of AIV at the given doses. The potent antiviral action of HHT may be restricted to a number of classes of RNA viruses.

Time dependencies were also studied. We detected that HHT treatment at 1000 nM produces an inhibition of viral yield of 3.0 and 4.6 orders of magnitude for DNA viruses HSV-1 and PRV, at 72 and 48 h.p.i., respectively. It is interesting to note that cells that were infected with HSV-1 or PRV for 10 h in the presence of HHT at 100 nM showed strong reduction in viral replication. These results indicate that HHT’s effect is time-dependent, and HHT should be utilized against DNA viruses in the earliest feasible stage of replication. Considering its selective antiviral activity, we hypothesize that HHT may sensitize cells to prevent viral replication through additional, complementary mechanisms.

Although viruses are completely dependent upon the host translational machinery, they use diverse mechanisms for translation initiation. Total eIF4E activity is required for proliferation of both tumor and normal cells, whereas phosphorylated eIF4E is not essential for normal cell proliferation and survival, but is specifically required for cancer cells [54,55,56]. Stimulation of eIF4E phosphorylation is correlated with facilitated translation and replication of some viruses [57,58,59], including murine coronavirus [60] and herpesvirus HSV-1 [61]. In contrast, dephosphorylation of eIF4E occurs during infection with influenza virus [62], VSV, or encephalomyocarditis virus (EMCV) [63]. Sometimes, phosphorylation is not required for eIF4E to support the viral replication of hepatitis E virus [64]. Notably, fibroblasts and mice in which the serine at position 209 of eIF4E is replaced with alanine (low level of p-eIF4E) are less susceptible to virus infections, including HSV-1, VSV, and EMCV [65]. Therefore, the phosphorylation status of eIF4E acts as a determinant of host susceptibility to a virus.

In the current study, HHT was found to attenuate the phosphorylation level of endogenous and exogenous eIF4E in accordance with viral proteins of PEDV, HSV-1, and PRV. These results demonstrated that the inhibitory effect of HHT on viral infections is associated with the phosphorylation of eIF4E. Phosphorylated eIF4E would rather favor the translation of selective mRNAs than impair translation initiation on a more global level upon viral infection [58,66]. Thus, it is promising to find possible therapeutic agents that are involved in selective translation, with viral replication specifically blocked but host cell translation unaffected. This research is, of course, far from complete, and it is worthy of further development.

HHT’s broad activity offered interesting perspectives for repurposing this anti-tumor drug as an antiviral. Compared to traditional drug discovery and development, a repositioned drug offers significant advantages, including minimal risk of failure, since the toxicity, tolerance, and pharmacokinetic properties of the drug are already known. Therefore, the cost and time needed to bring a drug to market are significantly reduced. Furthermore, short duration therapy—several days are sufficient to treat most acute viral infections, while chronic diseases might require months or years—would presumably reduce drug loading, and therefore, drug-related toxicity and possible side effects. The broad inhibitory effect of HHT on viral replication warrants further evaluation of this natural compound as a first-line therapeutic to combat viruses, including emerging and pathogenic species. Combination therapy to target both viral components and cellular mechanisms might improve antiviral efficacy, reduce viral resistance, and minimize toxicity and side effects in the control of viral infections and epidemic viral diseases.

## 5. Conclusions

In conclusion, HHT potently inhibited the viral replication of VSV, NDV, PEDV, HSV-1, and PRV; by comparison, HHT treatment moderately inhibited infection by AIV at the given doses. Furthermore, HHT-treated embryos, chickens, and piglets were less susceptible to viral infections of NDV, NDV, and PEDV. In addition, our preliminary results show that HHT antagonizes the phosphorylation level of endogenous and exogenous eIF4E in Vero and HeLa cells. The potent antiviral action of HHT is restricted to a number of classes of viruses; more details and clarification are still needed.

## Figures and Tables

**Table 1:** Antiviral activity of ribavirin, acyclovir, quercetin, and homoharringtonine (HHT).

Cells	Test Drugs	Virus	CC_50_^a^	IC_50_^b^	SI ^c^
HeLa	HHT	NDV	1.918	0.018	107
HeLa	Ribavirin	NDV	2821	44.241	64
Vero	HHT	PEDV	5.582	0.112	50
Vero	Quercetin	PEDV	>1000	6.897	>145
Vero	HHT	HSV-1	5.582	0.139	40
Vero	Acyclovir	HSV-1	3809	0.789	4828

Experiments were carried out in triplicate. ^a^ half maximal cytotoxic concentration (µM). ^b^ half maximal inhibitory concentration (µM). ^c^ Selectivity index = CC_50_/IC_50_.
